# Aberrant activation of the Hedgehog signaling pathway in granulosa cells from patients with polycystic ovary syndrome

**DOI:** 10.1080/21655979.2021.2003943

**Published:** 2021-12-07

**Authors:** You Li, Guohui Xiong, Jun Tan, Shudi Wang, Qiongfang Wu, Lei Wan, Ziyu Zhang, Ouping Huang

**Affiliations:** a Reproductive Medicine Center, Jiangxi Maternal and Child Health Hospital, Nanchang, Jiangxi, China; b Department of Orthopaedics, Nanchang Hongdu Hospital of Traditional Chinese Medicine, Nanchang, Jiangxi, China; c Nanchang University, Nanchang, Jiangxi, China; d Key Laboratory of Women’s Reproductive Health of Jiangxi Province, Jiangxi Maternal and Child Health Hospital, Nanchang, Jiangxi, China; e Department of Pathology, Jiangxi Maternal & Child Health Hospital, Nanchang, Jiangxi, China

**Keywords:** PCOS, hedgehog, Ihh, Ptch2, Gli

## Abstract

The molecular mechanism that triggers polycystic ovary syndrome (PCOS) is mysterious. Abnormal development of ovarian granulosa cells (GCs) is one of the causes of PCOS. Herein, our study was carried out using RNA-seq to detect the different gene expression levels in ovarian GCs between three patients with PCOS and four normal controls. To verify the RNA-seq data, GCs from 22 patients with PCOS and 21 controls with normal ovulation were collected to perform the RT-PCR analysis. Hedgehog signaling pathway (Hh) members, Ihh and Ptch2 were abnormally highly expressed in the PCOS tissue (PT). The qPCR also indicated that the expression levels of Hh signaling pathway downstream members, Ptch1, Gli1, and Gli2 in the PT were significantly higher than those in the normal tissue (NT). Besides, the expression of TNF-α mRNA in PCOS patients was higher than that in the control group. Through the chromatin immunoprecipitation assay (ChIP), we found that the Gli1-IP-DNA enriched from the granular cells of PCOS patients was higher than that of the control group. Finally, the Hh signaling pathway inhibitor, cyclopamine, can decrease the apoptosis of PCOS ovarian granulosa cells. These results suggest that abnormal activation of Hh signaling pathway, especially Ihh signal, may have a profound influence on PCOS.

## Introduction

Polycystic ovary syndrome (PCOS) is one of the most common endocrine disorders in women, which affects 10% women of reproductive age [1,2]. The Rotterdam Criteria requires women to conform to two of three following symptoms: oligo-ovulation or anovulation, clinical or biochemical hyperandrogenism and polycystic ovaries (PCO) [3]. In the late stage of PCOS, the complication associated with metabolic diseases such as diabetes, abdominal adiposity, high cholesterol and hypertension mostly occur [4–6]. Therefore, the PCOS patients need a long-term therapy to avoid adverse outcomes. But the etiology, special molecular mechanism and pathogenesis are still unclear.

Folliculogenesis, a highly coordinated event in the development and release of oocytes, is disrupted in PCOS. In PCOS women, excessive primordial follicles were recruited and subsequent development was arrested at the early preantral stage, resulting in the formation of multiple cysts [7]. Increased GnRH pulses favor LH production, which along with excess insulin stimulates ovarian theca cells to produce more androgen resulting in cessation of follicular growth and dominant follicle selection, thus affecting normal ovulation. Therefore, one of the most important symptoms of PCOS is the disorder of follicular development. However, the precise molecular defect in follicular development in PCOS remains unknown. Some studies have found that the development of PCOS may be related to miRNA, cicrRNA and inflammatory signaling pathways [8–11]. Follicle development is precisely regulated by the communication between the oocyte, granulosa (GCs) and theca cells [12–14], involving several signaling pathways [15–19]. Studies have reported that when the growth and differentiation factor GDF9 secreted by oocytes is lacking, the theca cells (TCs) layer cannot be formed, the production of steroids is reduced, the follicle is immature, and the corpus luteum cannot be formed [20,21]. Subsequent studies have shown that this phenomenon is due to the lack of GDF9, which leads to a decrease in the secretion of hedgehog (Hh) ligands (mainly Dhh and Ihh) from GCs, making it unable to interact with Ptch receptors on the cell membrane of TCs adjacent to GCs through paracrine, resulting in the Hh signal pathway fails to transduct normally, and the TCs layer forms obstacles, which eventually leads to abnormal follicular development [22]. Therefore, under normal circumstances, GCs can transmit the Hh ligand they secrete to the Ptch receptor on TCs through the paracrine pathway, thereby transducing and regulating the activity of the Hh pathway and the normal development of TCs [22].

The *hedgehog* gene was first cloned in Drosophila [23]. It plays a very important role in the embryonic development and remodeling of adult tissues [24]. Hh signaling regulates cell fate determination, proliferation and differentiation [25]. Moreover, over-activation of Hh signaling is associated with the tumorigenesis [26–29]. In mammals, the Hh pathway consists of three Hh ligands, indian (Ihh), desert (Dhh), and sonic (Shh). The membrane receptors are patched (Ptch1,2) and transmembrane signal transducer protein smoothened (SMO) [30]. In the absence of Hh ligand, Ptch maintains SMO in an inactive state. Binding of Hh ligand to Ptch relieves inhibition of SMO, and allows the activation of Hh-induced intracellular transcriptional effectors Glioma-associated oncogene homolog (Gli-1, 2, 3), leading to the induction of target gene expression [31].

Recently, expression of components in the Hh pathway was observed in both GCs and residual ovarian tissue, and the expression was changed in response to the stages of follicular development in human postnatal ovaries [32]. Based on this, in order to explore whether the Hh signaling is abnormally activated in the GCs of PCOS patients, we plan to use RNA-seq and functional analysis to verify it, in order to discover the important role of the Hh pathway in the pathogenesis of PCOS.

## Materials and methods

### Patients selection

A prospective case-control study and qPCR analysis were designed including 22 patients with PCOS group and 21 patients with regular menstrual cycles (control group) from December 2016 to March 2017 at Jiangxi Maternal and Child Health Hospital. In addition, RNA-seq analysis was performed on the ovarian granular cell layer tissues from three PCOS patients and four controls. 1) PCOS group inclusion criteria: 1 year ≤ years of infertility ≤ 20 years; 20 years ≤ age ≤ 35 years. Body Mass Index (BMI) 18–25 Kg/M2. The diagnosis of PCOS refers to the Rotterdam criteria and the Chinese PCOS diagnostic criteria, including sparse ovulation or anovulation and B-ultrasound showing polycystic ovarian-like changes, with or without symptoms or clinical manifestations of hyperandrogen. Oral glucose tolerance test (Oral glucose tolerance test, OGTT) test to check blood sugar and insulin, abnormal patients take the medicine normally and then start. 2) Inclusion criteria for the control group:1 year ≤ years of infertility ≤ 20 years; 20 years ≤ age ≤ 35 years. BMI 18–25 Kg/M2. The cause of infertility is male factors or fallopian tube problems. The menstrual cycle is 26–32 days, there is normal ovulation, the ovaries are non-PCO, and the number of eggs retrieved is ≥5. On the third day of the menstrual cycle, serum follicle-stimulating hormone (FSH)≤10mIU/mL, luteinizing hormone (LH)≤10mIU/mL and estradiol (Estradiol, E2)≤50 pg/mL. The OGTT test checks that blood sugar and insulin are normal. 3) Exclusion criteria for patients: previously performed one-sided oophorectomy was diagnosed with uterine abnormalities. Uterine malformations (such as unicornuate uterus, mediastinal uterus, bicornuate uterus, double uterus, etc.), endometriosis, adenomyosis, uterus fibroids, intrauterine adhesions and chromosomal abnormalities in either spouse. Three or more recurrent miscarriages (including biochemical pregnancy miscarriage), abnormal thyroid function, hyperprolactinemia and other endocrine diseases are untreated. Patients with contraindications to assisted reproductive technology or pregnancy. If there is hyperandrogenemia, other causes of hyperandrogenemia should be excluded, such as congenital adrenal hyperplasia, Cushing’s syndrome, androgen-secreting tumors, 21-hydroxylase deficiency atypical adrenal hyperplasia, and so on.

### Blood sampling and hormone measurement

Blood samples were collected on the third day of the menstrual cycle and on the day of HCG injection, subsequently centrifuged at 4000x g for 1 min. The serum was used for the quantitative determination of hormone (follicle-stimulating hormone (FSH), luteinizing hormone (LH), estradiol (E_2_), prolactin (PRL) and testosterone (TES) level by chemiluminescent enzyme immunoassay using Automated Enzyme Immunoassay Analyzer (AIA-2000ST, TOSOH CORPORATION). The above measurements were repeated three times, and the results come from the clinical test results.

### Human GCs collection and cell culture

For ovarian stimulation, follicular aspirates were collected during oocyte retrieval following published procedures and ovarian stimulation was the use of a prolonged protocol [33,34]. Briefly, gonadotropin-releasing hormone agonist (GnRH-a, Ipsen, Boulogne-Billancourt, France) was used in the second or third day of menstrual cycle for pituitary down-regulation. Gonadotropin stimulation was started after 28 or 38 days following the criteria: no ovarian cysts > 8 mm, E_2_ < 50 pg/ml, FSH < 5 IU/L, LH < 5 IU/L. Initially, patients received 75–112.5 IU/d of recombinant human FSH (Merck-Serono, Darmstadt, Germany) according to the patient’s age, body mass index (BMI), serum basal FSH levels, LH levels, E_2_ levels and antral follicle count. The time and dose of recombinant human FSH were adjusted according to ovarian response as monitored by serum E_2_ levels and vaginal ultrasound. When the dominant follicle was ≥19 mm in diameter or at least three follicles were ≥17.5 mm in diameter, recombinant human FSH was stopped, and a single injection of 6000–8000 IU of hCG (Merck-Serono, Darmstadt, Germany) was administered. Oocytere-trieva l was performed 36–40 h later under transvaginal ultrasound guidance.

Follicular fluids were centrifuged at 2000 rpm for 5 min. The cells were resuspended with DMEM/F 12 (Life Technologies, Carlsbad, CA, USA) medium and transferred to a 50% (volume fraction) Percoll gradient (Sigma-Aldrich, Germany). They were centrifuged at 4000 rpm for 20 min to purify human GCs from any red blood cells. After washing and recentrifugation, sheets of human GCs were digested with trypsin at a 1:1 ratio for 4 min to separate them. The GCs were removed using a pipette and washed with phosphate buffered saline (PBS). 10% FBS DMEM/F12 culture medium at a 2:1 ratio was added to terminate the digestion, centrifuged at 1500 rpm for 3 min. GCs were resuspended with 1xPBS at a 1:5 ratio, centrifuged at 1500 rpm for 3 min. After discarding the supernatant, added 1 ml of 10% FBS DMEM/F12 culture medium, resuspended, inoculated in a dish, and placed in a 37°C, 5% CO2 incubator. After observing the morphology of the granulosa under the microscope, changed the fluid per 24 h. The cells at indicated time were stored at −80°C for future analysis.

### FSHR (follicular stimulating hormone receptor) immunohistochemical staining in human GCs

The immunohistochemical staining in cells was carried out as described previously [35]. After the GCs are cultured for 3 days, the fixed cell slides are washed with PBS for 3 times, and fixed with 4% paraformaldehyde for 30 min, washed with PBS for 3 times. Incubated in 3% H_2_O_2_ deionized water for 10 min to block the effect of endogenous peroxidase, washed in 1xPBS for 2 times. Drop anti-rabbit anti-human FSHR polyclonal antibody (1:100) dropwise at 4°C overnight, washed in PBS for 2 times. Added reagent 1 (polymer helper) dropwise, incubated at room temperature for 20 min, and washed in 1xPBS for 2 times. Added reagent 2 (polyperoxidase-anti-mouse/rabbit IgG) dropwise and incubated at room temperature. Finally, DAB was added dropwise for color reaction (control reaction time under microscope). Rinsed thoroughly with tap water, counterstained with hematoxylin for 1 min, dehydrated with conventional gradient alcohol, transparent xylene and seal with neutral resin. As a negative control, 1xPBS was used instead of the primary antibody. The cells with yellow staining on the cell membrane were FSHR positive cells, and the number of positive cells in 10 high-power (× 400) visual fields was randomly counted.

### RNA-seq and qPCR

RNA isolation was carried out as described previously [36]. Total RNA was extracted from four normal tissues and three PCOS tissues using the RNAiso reagent (TaKaRa, Shiga, Japan). The library was validated on the Agilent Technologies 2100 bioanalyzer subjected to deep sequencing on Illumina HiSeq 2000 (50-bp single-read sequencing), and analyzed at BGI Genomics Co., Ltd. For RT-qPCR analysis, the GCs total RNA from 22 patients with PCOS and 21 controls were carried out using the PrimeScript RT reagent Kit (TaKaRa). Standard qPCR was carried out with the following primers: hsa-RT-Ptch1 (5ʹ-GCTGCACTACTTCAGAGACTGG-3ʹ and 5ʹ-CACCAGGAGTTTGTAGGCAAGG-3ʹ), hsa-RT-Gli1 (5ʹ-AGCCTTCAGCAATGCCAGTGAC-3ʹ and 5ʹ-GTCAGGACCATGCACTGTCTTG-3ʹ), hsa-RT-Gli2 (5ʹ-GTCAGAGCCATCAAGACCGAGA-3ʹ and 5ʹ-GCATCTCCACGCCACTGTCATT-3ʹ), hsa-RT-Gli3 (5ʹ-TCAGCAAGTGGCTCCTATGGTC-3ʹ and 5ʹ-GCTCTGTTGTCGGCTTAGGATC-3ʹ) and has-RT-actin (5ʹ-ACCTTCTACAATGAGCTGCG-3ʹ and 5ʹ-CCTGGATAGCAACGTACATGG-3ʹ). Real-time PCR was carried out using the FastStart SYBR Green Master mix (Roche) on a 7500 Real-Time PCR System (Applied Biosystems, Grand Island, NY). The actin was used as an internal control. The results were presented as fold changes, calculated using the 2^−ΔCT^ method, and a ratio of expression in the PCOS relative to the Controls less than 1.0 was considered to be low.

### Apoptosis analysis

The apoptosis analysis was carried out as described previously [37]. 2–3 portions of human ovarian granulosa cells from PCOS patients were mix and culture for 48 hours, then were treated with Smo inhibitor KAAD-cyclopamine (CPA, 1um) for 24 hours. Collect the cells by centrifugation, discard the supernatant, and wash the cells twice with pre-cooled PBS. Add pre-cooled 70% ethanol, fix overnight at 4°C. Staining and centrifugation to collect the cells, wash the cells once with 1 ml of 1xPBS, add 500ul PBS containing 50ug/mL ethidium bromide (PI), 100ug/mL RNase A, 0.2% Triton X-100, incubate at 4°C in the dark for 30 minute. Flow cytometry detect with a flow cytometer according to standard procedures, generally count 2–3 million cells, and analyze the results with the cell cycle simulation software CXP.

## Results

We first performed transcriptome sequencing and differential gene expression analysis on granule cells in IVF-ET treatment of PCOS patients in order to find the signal pathways with changes. Later, we plan to use molecular biology and cell function analysis to detect the role of pathway changes in GCs.

### Both Ihh and Ptch2 were upregulated in PCOS

**Figure 1.**
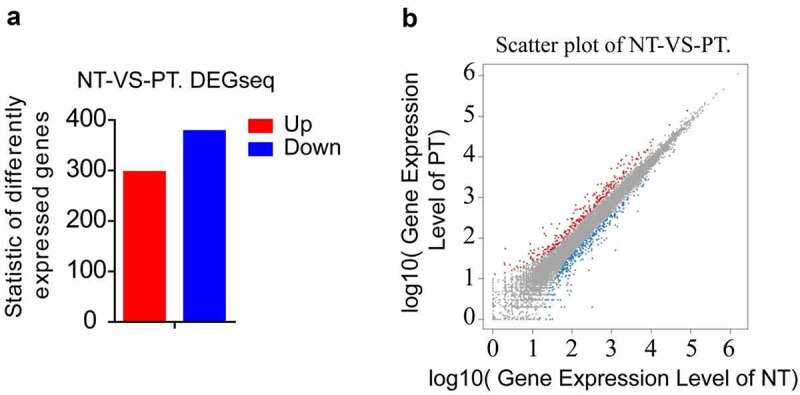
**Summary of DEGs by RNA-seq**. (a) X axis represents comparison method between each group. Y axis represents DEG numbers. Red color represents up-regulated DEGs. Blue color represents down-regulated DEGs. (b) Scatter plot of DEGs. X Y axis represents log10 transformed gene expression level, red color represents the up-regulated genes, blue color represents the down-regulated genes, gray color represents the non-DEGs

**Figure 2.**
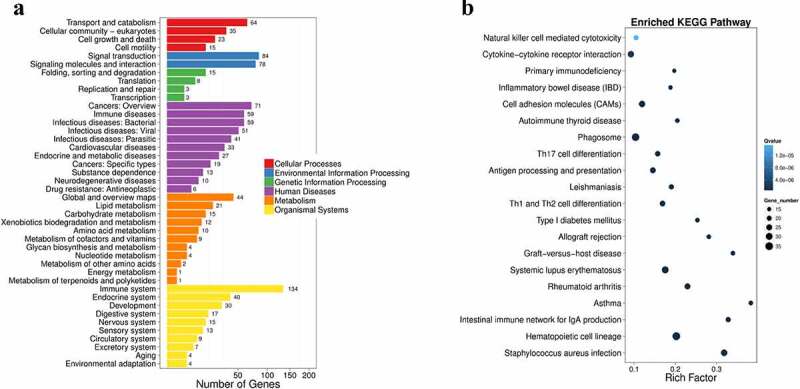
**Pathway functional enrichment results of NT-VS-PT. DEGseq Pathway**. (a) X axis represents number of DEG. Y axis represents functional classification of KEGG. There are seven branches for KEGG pathways: Cellular Processes, Environmental Information Processing, Genetic Information Processing, Human Disease (For animals only), Metabolism, Organismal Systems and Drug Development. (b) X axis represents enrichment factor. Y axis represents pathway name. The color indicates the q-value (high: white, low: blue), the lower q-value indicates the more significant enrichment. Point size indicates DEG number (The bigger dots refer to larger amount). Rich Factor refers to the value of enrichment factor, which is the quotient of foreground value (the number of DEGs) and background value (total Gene amount). The larger the value, the more significant enrichment

In order to explore the molecular mechanism of PCOS, we used RNA-seq analysis to detect the difference in gene expression levels between the PCOS group (PT) and the normal tissue (NT). The RNA-seq data showed that there were total 673 differentially expressed genes (DEG). Among them, 296 genes were up-regulated and 377 genes were down-regulated (Figure 1). Moreover, the pathway functional enrichment results of NT-VS-PT. DEGseq pathway indicated that immune system-related genes occupied an important part of DEG (Figure 2). Interestingly, among DEGs, we found that the Hh pathway member, ligand Ihh and the receptor Ptch2 (also a target gene downstream of the Hh pathway) are highly expressed in the PT (Table 1). Next, we would use more clinical samples for subsequent analysis.

**Table 1.** Expression of a part of DEGs hierarchical clustering analysis results of PCOS group and control group

Symbol	GeneID	Length	NT	PT	log2(PT/NT)	Up/Down	*p*-Value
TNXA	7146	2783	168.4	354.2	1.4	Up	<0.001
IL4I1	259,307	1798	168.2	350.4	1.4	Up	<0.001
S100B	6285	1135	357	572	1.0	Up	<0.001
**IHH**	3549	2074	20	129	3.0	Up	<0.001
APOD	347	1148	878	1984	1.5	Up	<0.001
SDS	10993	1620	410	1322	2.0	Up	<0.001
**PTCH2**	8643	3840	1803.9	3109.2	1.1	Up	<0.001
CTSH	1512	1532	1616	2873	1.2	Up	<0.001
LILRB5	10,990	2395	361.4	1231.8	2.1	Up	<0.001
CCL4	6351	667	2042.2	3323.8	1.1	Up	<0.001
C4B	721	5444	838.9	1840.7	1.5	Up	<0.001

### General conditions

The results of general comparison between the PT and NT of patients in Table 2 showed that there was no significant difference in the age, infertility, BMI, blood test FSH, E2, and PRL in the PT compared with control group. On the other hand, the LH and TES serum concentrations in the PT were increased significantly (P ≤ 0.05) compared with control group.

**Table 2.** Comparison of general conditions between PCOS group and control group

Characteristics	PCOS (n = 22)	Control (n = 21)	*P*
Age (years)	27.64 ± 2.82	28.95 ± 2.77	>0.05
Infertility duration (years)	4.05 ± 2.44	3.14 ± 2.29	>0.05
BMI (Kg/m^2^)	22.03 ± 3.25	22.67 ± 3.47	>0.05
Basal FSH (IU/L)	5.42 ± 1.09	5.93 ± 1.36	>0.05
Basal LH (IU/L)	7.30 ± 3.59	4.43 ± 2.35	<0.05
Basal E_2_ (pg/mL)	39.05 ± 18.67	40.11 ± 17.00	>0.05
PRL (ug/L)	19.02 ± 13.24	17.65 ± 9.84	>0.05
T (nmol/L)	38.28 ± 20.30	26.34 ± 10.79	<0.05

Note: The date were expressed asMean ± SD.

### Clinical outcomes

In the general comparison of IVF-ET treatment between the two groups of patients, the endometrial thickness of HCG on the PT (10.6 ± 1.7 mm) was significantly lower than that of the control group (11.9 ± 2.0 mm). Other indicators such as total Gn amount, total Gn days, HH day LH, E2 and P were not statistically significant in the two groups. The MII egg rate of the PT (60.7 ± 28.9%) was significantly lower than that of the control group (80.4 ± 13.6%), and the two groups were statistically significant. The 2 PN fertilization rate of the PT (61.7 ± 20.8%) was significantly lower than the control group (76.9 ± 18.6%), the two groups were statistically significant. Other indicators such as the number of eggs obtained, the number of embryos available and the rate of superior embryos were not statistically significant between the two groups (Table 3).

**Table 3.** Comparison of clinical outcomes between PCOS group and control group

Variables	PCOS	control	*P*
Dosage of Gn (IU)	1916 ± 944	2144 ± 925	>0.05
Duration of Gn (days)	12.7 ± 2.6	11.6 ± 1.7	>0.05
Endometrial thickness on HCG day (mm)	10.6 ± 1.7	11.9 ± 2.0	<0.05
LH on HCG day (IU/L)	1.1 ± 0.6	0.9 ± 0.5	>0.05
E_2_ on HCG day (pmol/l)	2269 ± 1155	2510 ± 1110	>0.05
P on HCG day (nmol/l)	1.1 ± 0.4	1.0 ± 0.5	>0.05
No of oocytes retrieved	16 ± 9	14 ± 6	>0.05
MII oocyte rate (%)	60.7 ± 28.9	80.4 ± 13.6	<0.05
Fertilization rate (%)	61.7 ± 20.8	76.9 ± 18.6	<0.05
Cleavage rate (%)	97.7 ± 5.9	92.7 ± 12.0.	>0.05
No of Embryo	3.4 ± 1.6	4.1 ± 2.4	>0.05
Good quality embryo rate (%)	2.1 ± 1.8	3.1 ± 2.8	>0.05

Note: The date were expressed as mean + SD.

### Expression of Hh family members in GCs from PT and control Group

**Figure 3.**
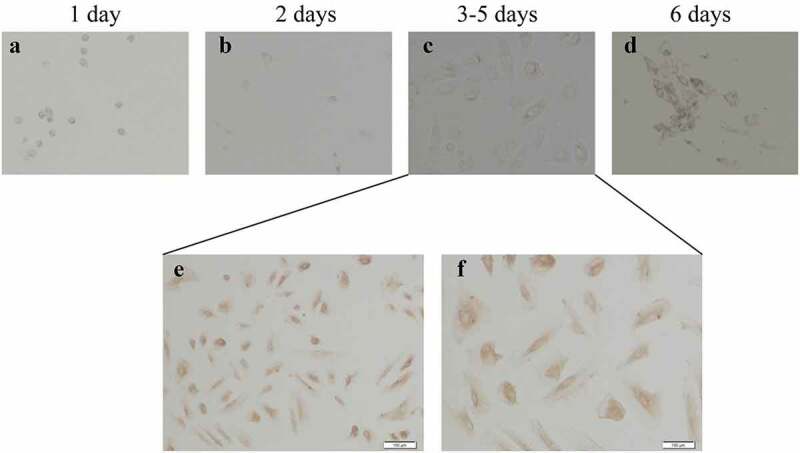
**Culture and identification of ovarian GCs in vitro**. (a)-(d) represent the situation of GCs cultured in vitro for 1–7 days. (e)-(f) represent the IHC results of GCs staining with FSH antibody during 3–5 days. Scale bar = 100 μm for (e), Scale bar = 50 μm for (f)

**Figure 4.**
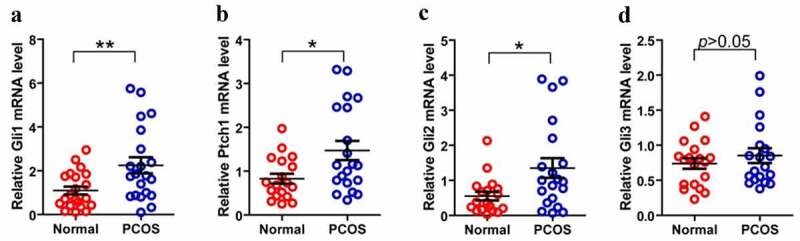
**Expression of Hh family members in GCs from PT and control Group**. (a)–(d) represent Gli, Ptch1, Gli2 and Gli3 mRNA levels form GCs between normal and PCOS. The red circle represents the normal group (Normal), and the blue circle represents the experimental group (PCOS). *p*-Values were determined by Student’s t-test, *p < 0.05, **p < 0.01

As we know that PCOS is often accompanied by abnormal follicular development, therefore, we consider that whether the disordered Hh signaling pathway is contributed to the PCOS-related abnormal follicles. Then we isolated and purified granulosa cells from 22 PCOS and 21 Control samples. We performed FSH staining after 3–5 days of cell culture (Figure 3). After identification, total RNA was extracted for qPCR detection. Next, we compared the mRNA levels of Gli1, Gli2, Gli3 and Ptch1 in PCOS and non-PT undergoing IVF treatment to explore the potential role of Hh signaling pathway in PCOS-related abnormal follicles. We tested the expression of Hh family members of GCs in both PCOS and controls. The levels of Gli1 mRNA, Ptch1 mRNA and Gli2 mRNA were significantly higher in PT than those in the control group, while the expression of Gli3 mRNA had no significant difference between the two groups (Figure 4). These results indicate that the abnormality of the Hh pathway has a potential role in the development of PCOS.

### The expression of TNF-α mRNA in PCOS patients was higher than that in the control group

**Figure 5.**
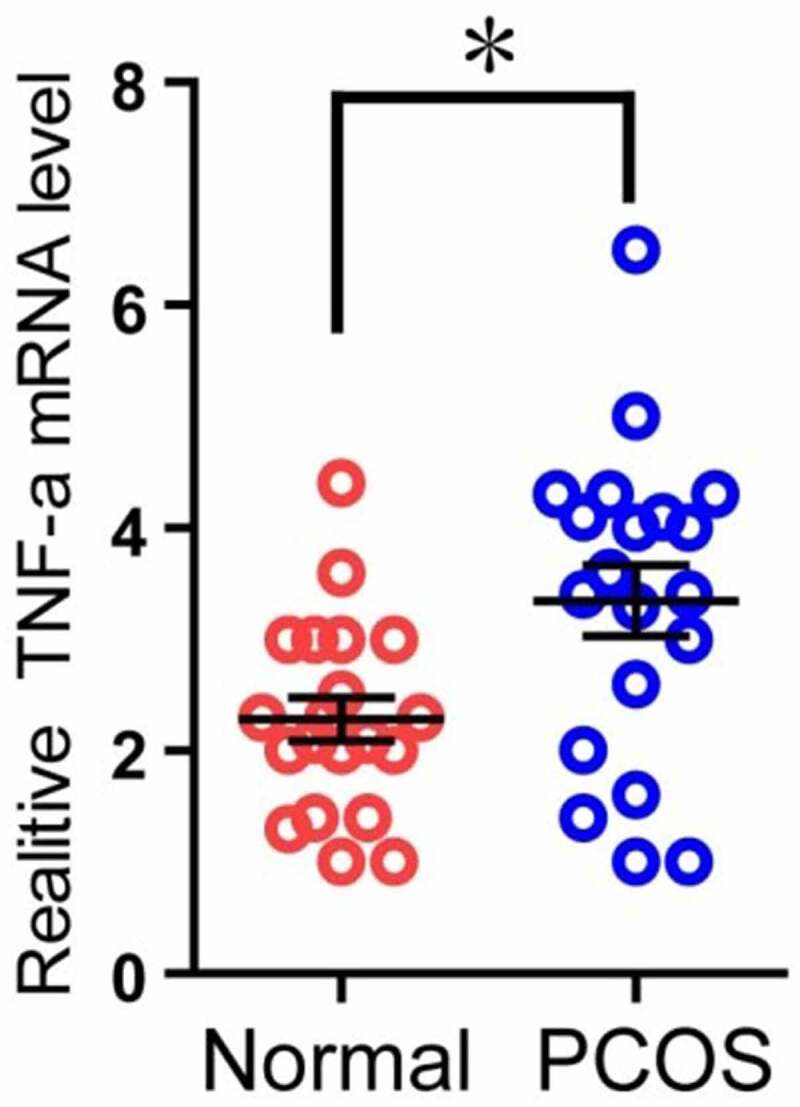
**Comparison of TNF-α expression in granulosa cells of two groups**. The relative difference of TNF-α mRNA expression results between the normal group (Normal) and the experimental group (PCOS). The red circle represents the normal group (Normal), and the blue circle represents the experimental group (PCOS). * *p* < 0.05

Because TNF-α can induce apoptosis, and the RNA-seq results also showed that the expression of TNF-α in the PT was higher than that in the control group (Table 4), and it was statistically significant. Therefore, we used the second part of the mRNA of the granulosa cells of the two groups of patients to perform qPCR experiments to verify the above RNA-seq data. The results showed that in the PT, the mRNA level of TNF-α was significantly higher than that of the control group, and it was statistically significant (Figure 5). The above results indicate that the expression of TNF-α mRNA in PCOS patients is higher than that in the control group.

**Table 4.** Expression of a part of DEGs hierarchical clustering analysis results of PCOS patients and control group

Symbol	GeneID	Length	NT	PT	log2(PT/NT)	Up/Down
54,209	TREM2	266	810	1.96	Up	<0.001
81,035	COLEC12	497	1119	1.52	Up	<0.001
**7124**	**TNF**	**848**	**1547**	**1.22**	**Up**	<0.001
23,237	ARC	407	973	1.61	Up	<0.001

### Inhibition of Hh signaling pathway can decrease the apoptosis of PCOS ovarian granulosa cells

**Figure 6.**
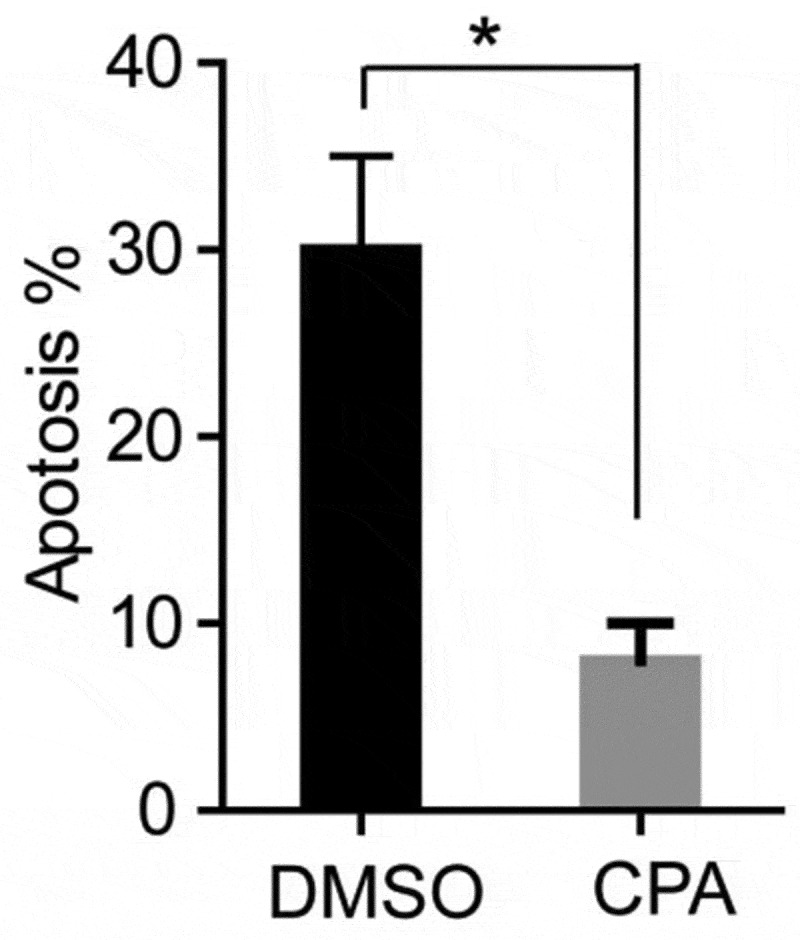
**CPA treatment of PCOS ovarian granulosa cells to detect its apoptosis**. PCOS cells were treated with KAAD-cyclopamine (CPA, 1um) 24 h later to detect the apoptosis, and then compared with the control group (DMSO). * *p* < 0.05

Because TNF-α can induce apoptosis, and TNF expression and Hh signal increase in GCs of PCOS patients (Figures 4 and 5), suggesting that the activation of Hh pathway may be related to the apoptosis of GCs. In order to detect whether excessive activation of the Hh signaling pathway can promote cell apoptosis, we used PCOS ovarian granulosa cells for apoptosis detection experiments. The cells were treated with KAAD-cyclopamine (CPA, 1um), an inhibitor of the Hh signaling pathway, for 24 hours, and then the cell apoptosis was detected by flow cytometry using annexin V-FITC and prodiduim iodine (PI) double staining. The results showed that, after CPA treatment, the apoptosis ratio of GCs was significantly reduced (Figure 6). The above results indicate that inhibition of Hh signaling pathway can simultaneously reduce the apoptosis of PCOS ovarian granulosa cell.

### TNF-α is a target gene of Hh signaling pathway

**Figure 7.**
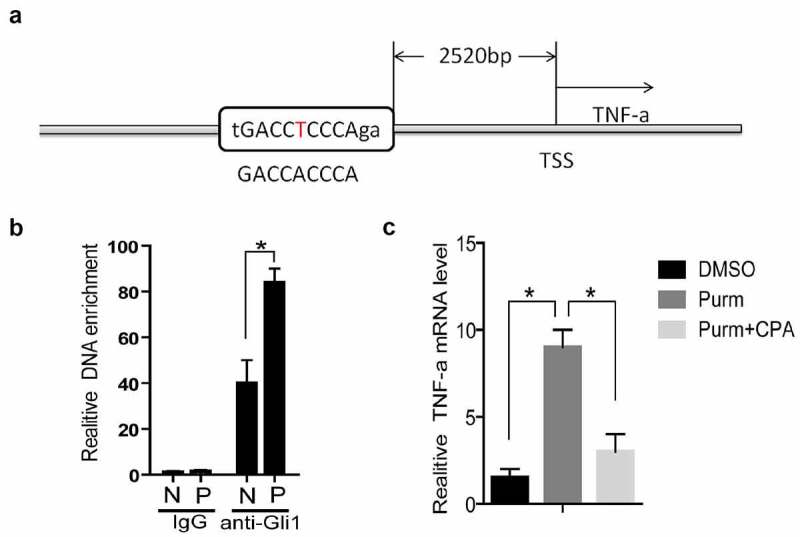
**TNF-α is a target gene of Hh signaling pathway**. (a) TSS stands for the transcription start site, and its upstream 2520 bases is the Gli binding site predicted by rVista2.0 software, with one base error (red T). (b) In the chromatin immunoprecipitation experiment (ChIP), the Gli1 antibody was used to detect the role of Gli protein in the predicted TNF-α promoter region Gli binding site in the normal group of cells and the experimental group of cells. IgG is the control, anti-Gli1 is immunoprecipitation of Gli1 antibody, N is the normal control group, and P is the experimental PCOS group. *represents *p* < 0.05. (c) The NIH3T3 cells were treated with DMSO, purm or purm+CPA for 24 hours, and TNF-α mRNA detection was performed. *Represents *p* < 0.05

The expression of Gli1 and Gli2 in PCOS is higher than that of the control group (Figure 4). As transcription factors, the Gli proteins can bind to a specific sequence of DNA promoter regions (GACCACCCA) to promote the transcriptional level of downstream genes [38,39]. We used rVista 2.0 software to predict [40] and found that there is a Gli binding site (Gli-BS) in the upstream of TNF-α (Figure 7(a)). By using chromatin immunoprecipitation assay (ChIP), it was found that the DNA enriched from the granular cells of PCOS patients was higher than that of the control group (Figure 7(b)). The above results indicate that Gli1 may have a regulatory effect on TNF-α. In order to confirm this phenomenon, we used purmorphamine (Purm, 10 μM), an agonist of the Hh signaling pathway receptor Smo, to treat NIH3T3 cells, and the mRNA level of TNF-α was increased (Figure 7(c)). Then the Smo inhibitor KAAD-cyclopamine (CPA, 1 μM) was used to treat NIH3T3 cells, and the mRNA level of TNF-α was found to decrease (Figure 7(c)). The above results prove that the Hh signaling pathway can stimulate the TNF-α mRNA expression level through the Gli protein.

## Discussion

In our research, PCOS patients were diagnosed based on the Rotterdam criteria including oligoovulation or anovulation and PCO. This study was a matching study and no statistically significant difference was found in age, infertile period, or BMI in comparing the two groups as we showed in Table 2. The basic LH and T levels of PT are high, which is abnormally consistent with its endocrine results [4,5,7]. Previous studies have found that PCOS patients were often accompanied with aberrant follicles and unsatisfactory fertilization rate [33,34,41]. We also analyzed the clinical outcomes of PCOS group and control group. As shown in Table 3, the MII oocyte rate and fertilization rate were remarkably reduced in PCOS group when compared with the control group. In line with previous studies, our data also showed a high rate of immature oocyte and low rate of fertilization in PCOS patients.

In our study, patients in both groups were treated with controlled ovarian hyperstimulation (COH) after down-regulation with an ultralong protocol. Studies have shown that prolonged down-regulation can significantly improve the thickness, morphology, and blood flow of the endometrium, thereby increasing the implantation rate and pregnancy rate [41]. At the same time, it can reduce the concentration of pelvic inflammatory cytokines and improve pelvic cavity. The environment is favorable for embryo implantation [42]. In our study comparing IVF-ET treatment in general, the endometrial thickness of HCG in the PT was significantly lower than that in the control group. Other indicators such as total Gn amount, total Gn days, HH day LH, E2 and P were not statistically significant in the two groups. Studies have shown that an appropriate thickness of the endometrium is important for embryo implantation. When the endometrial thickness is >14 mm on the day of hCG injection, the IVF implantation rate and pregnancy rate are reduced, and the endometrial thickness is <6. At ~7 mm, the embryo implantation rate and pregnancy rate are affected, and the miscarriage rate increases. Besides, the average endometrial thickness of PCOS patients on HCG day was 10.6 mm, lower than the NT’s 11.9 mm, were within the normal range and had no obvious clinical significance.

In IVF-ET treatment, the MII egg rate is the ratio of mature eggs. After the sperm and the egg meet, the sperm enters the egg, and the nucleus of the egg and the nucleus of the sperm will form a pronucleus, respectively. The two pronucleus are called ‘2 PN’, indicating that the egg is fertilized normally. The 2 PN fertilization rate represents the rate of normal fertilization of the egg. In our study, the MII egg rate of the PT was significantly lower than that of the control group, which was statistically significant. The 2 PN fertilization rate of the PT was significant was lower compared with the control group, the two groups were statistically significant, consistent with the results of previous studies of PCOS. At the same time, other indicators such as the number of eggs obtained, the number of available embryos, and the rate of superior embryos were also consistent with other studies. There was no statistical significance in the two groups.

The three Hh ligands (Ihh, Shh and Dhh), the two receptors (Ptch1 and Ptch2) and the mediator of Hh signaling (SMO) are expressed in GCs and in corpora lutea from pseudopregnant mice [32]. In addition, the transcription factor, Gli1, Gli2 and Gli3 are expressed in all ovarian tissues [43–45]. The ovarian Hh signaling system could be involved in the proliferation of GCs under certain conditions [46]. Expressions of a number of Hh genes in GCs that are known to be important for ovulation made no difference between mutants and controls [44]. Some studies suggest that an association exists between modulation of the Hh pathway and selection of the dominant follicle(s) [45]. In order to discuss the relationship between Hh signaling activity and PCOS which were accompanied with follicular dysplasia, we measured the components of the Hh pathway in GCs. We found that PT showed higher mRNA levels of Ptch1, Gli1 and Gli2, when compared to control groups; while the level of Gli3 mRNA had no significant difference. Ptch1 is a key component of the Hh signaling pathway, which controls cell fate determination during development [47]. Ptch1 mutations cause derepression of target genes, cell fate changes, and excessive growth in some tissues [48]. Gli1 lacks the N-terminal repressor domain and functions exclusively as an activator. The Gli1 gene is also a target of Hh signaling and thus acts to amplify the response to the signal [44,49]. Thus, the higher mRNA levels of Ptch1 and Gli1 confirmed that Hh signaling pathway is aberrant activated in the GCs of PCOS patients than control. In addition, Gli2 and Gli3 proteins contain both activator and repressor domains and undergo proteasome-dependent proteolytic cleavage [24]. In our research, although Gli3 acting mainly as a repressor was no difference in GCs from these two groups, Gli2 appearing to function mostly as an activator was significantly higher in GCs of PCOS.

Admittedly, the communication between oocytes and GCs is important for the development of follicular, in which GCs secrete various kinds of nutritional factors to promote oocyte growth, simultaneously, oocytes produce several factors to regulate GCs development [12,13,50]. Therefore, the function status of GCs is often considered as the mirror of oocyte quality. It is well known that PCOS patients are often accompanied with aberrant GCs in follicles [12–14]. The Hh signaling pathway was more activated in GCs of PCOS than control, which implied the aberrant activation of Hh signaling pathway was related to abnormal follicular development in PCOS patients.

## Conclusions

Herein, we first demonstrate that aberrant activation of Hh signaling pathway in GCs is related to abnormal follicular development in PCOS patients. These findings provide a basis for future investigations to define cell-specific response to Hh signaling in the follicle and to determine how the pathway modulates follicle development.

## Data Availability

The datasets used and/or analyzed during the current study are available from the corresponding author on reasonable request.

## Abbreviations

PCOS: polycystic ovary syndrome

GCs: granulosa cells

Hh: Hedgehog

SMO: smoothened

FSH: follicle-stimulating hormone

LH: luteinizing hormone

E_2:_estradiol

PRL: prolactin

TES: testosterone

GnRH-a: gonadotropin-releasing hormone agonist

PT: PCOS group

NT: Normal control group
